# Feeding *Pinus taeda* hydrolyzed lignin to dairy cows during *peripartum*: effects on health status, milk, and colostrum

**DOI:** 10.1016/j.vas.2026.100795

**Published:** 2026-08-01

**Authors:** Lucrezia Forte, Pasquale De Palo, Elisabetta Casalino, Tiziana Latronico, Grazia Maria Liuzzi, Aristide Maggiolino

**Affiliations:** a Department of Veterinary Medicine, University of Bari, 70010, Valenzano, BA, Italy; b Department of Biosciences, Biotechnologies and Environment, University of Bari, 70126, Bari, Italy

**Keywords:** Dairy cows, Polyphenols, Transition period, Peripartum, Oxidation

## Abstract

This study evaluated the effects of dietary supplementation with *Pinus taeda* hydrolyzed-lignin (Oxyphenol®) during the *peripartum* period on systemic metabolism, oxidative status, and milk and colostrum in dairy cows. A total of 20 Brown Swiss dairy cows, fed the same total mixed ration, were randomly assigned to two experimental groups (n = 10/grp): a control group and a group receiving Oxyphenol® from the beginning of the dry period until 60 days *postpartum*. The levels of serum biochemical profile markers remained largely within physiological ranges, indicating no adverse effects on systemic health. Oxyphenol® supplementation was associated with lower serum concentrations of alanine aminotransferase and alkaline phosphatase compared with controls, suggesting a possible modulation of hepatic-related biochemical indicators, although specific markers of liver function and inflammation were not assessed. Parallel reductions in circulating thiobarbituric acid reactive substances indicated alleviation of oxidative–metabolic strain around calving with Oxyphenol® treatment. The antioxidant effect was further supported by lower intracellular reactive oxygen species production and greater viability in peripheral blood mononuclear cells from Oxyphenol® cows, reflecting attenuated oxidative burden at the cellular level. In milk, Oxyphenol® did not affect gross composition, fatty acid profile, or coagulation traits, but increased antioxidant capacity and reduced lipid oxidation products, indicating improved oxidative stability. Colostrum composition was unaffected, though lipid peroxidation markers were reduced. These findings are compatible with improved maternal and mammary redox status; however, direct transfer of phenolic compounds or metabolites into milk and colostrum was not assessed and should be confirmed by targeted metabolomic studies.

## Highlights

• Pinus taeda hydrolyzed lignin was fed from dry-off to 60 days in milk.
• Oxyphenol® reduced blood lipid peroxidation around calving.
• PBMC from supplemented cows showed lower ROS and higher viability.
• Milk and colostrum composition were not modified by supplementation.
• Milk and colostrum showed improved oxidative stability.

## 1 Introduction

The transition period, commonly defined as the interval from the onset of the dry period to approximately three weeks after parturition, represents one of the most physiologically demanding phases for dairy cows (Pascottini et al., 2020). During this time, cows undergo extensive metabolic, endocrine, and immune adjustments that support the onset of lactation. A major consequence of the higher metabolic rate around parturition is the increased generation of reactive oxygen species (ROS). When ROS production exceeds the antioxidant capacity of the animal, oxidative stress develops. This redox imbalance can damage cellular structures, impair hepatic metabolism, and weaken immune cell function (Nakov et al., 2016). Concurrently, the immune system experiences a transient but marked suppression, affecting both innate and adaptive responses, and is characterized by reduced neutrophil activity, impaired lymphocyte proliferation, and altered cytokine production (Trevisi et al., 2012). The tight interplay between metabolic, endocrine, and immune functions during the transition period highlights the need for integrated nutritional and management strategies. In particular, nutritional approaches aimed at enhancing antioxidant defenses, supporting liver function, and modulating immune responses are increasingly recognized as key tools to improve the health and resilience of transition cows (Lopreiato et al., 2020). In this context, plant-derived bioactive compounds, particularly polyphenols, are gaining attention as promising nutritional strategy (Lopreiato et al., 2020). Owing to their well-documented antioxidant, anti-inflammatory, and immunomodulatory properties, dietary polyphenols may help mitigate ROS-induced damage, preserve cellular membrane integrity, and enhance immune competence during this critical phase (Benchaar et al., 2007; Santos et al., 2010). Beyond their systemic effects, polyphenols may also exert important actions at the rumen level. These compounds can modulate ruminal fermentation patterns, contributing to pH stabilization and reducing the risk of subacute ruminal acidosis through changes in microbial activity and decreased lactic acid accumulation (Jouany, 2006). Moreover, certain phenolic compounds have been reported to reduce methanogenesis, thereby improving feed efficiency and limiting energy losses (Drong et al., 2016).

Another aspect of growing interest concerns the potential transfer of dietary polyphenols, or more frequently their rumen-derived metabolites, into milk. Although this transfer is regulated by multiple factors and remains only partially understood, accumulating evidence indicates that phenolic compounds can be detected in milk, where they may enhance oxidative stability and potentially provide added functional value for human consumption (Forte et al., 2026).

Based on this background, the present study evaluated the effects of a polyphenol-rich additive, Oxyphenol®, derived from lignin obtained through hydrolysis of *Pinus taeda* bark. Oxyphenol® has previously been tested both in *vitro* and in *vivo* in beef cattle, where it showed promising results, including reduced enteric methane emissions (Maggiolino et al., 2019), improved immune and oxidative status (Maggiolino et al., 2020), and enhanced meat oxidative stability, particularly during a dry aging period (Maggiolino et al., 2021). Therefore, the objective of this study was to assess the effects of Oxyphenol® supplementation in the diet of dairy cows during the transition period, with specific focus on animal health, oxidative and immune status, and the potential impact on the composition and oxidative profile of colostrum and milk.

## 2 Materials and methods

### 2.1 Experimental design

This trial was approved by the Ethics Committee of the Department of Veterinary Medicine, University of Bari (protocol no. 07/2023). The experimental activities were carried out on a commercial dairy farm located in Southern Italy, starting on December 19, 2023, and ending on July 30, 2024. A total of 20 Brown Swiss dairy cows were enrolled and allocated into two experimental groups, balanced according to parity (CON = 2.9 ± 0.7; ADD = 2.8 ± 0.8): a control group (CON, *n* = 10) and a group receiving Oxyphenol® supplementation (ADD, *n* = 10). All animals received the same feeding regimens, with the diet adjusted according to the production phase: one diet from -60 to -21 days relative to calving, a second from -21 to +7 days, and a third from +7 to +60 days postpartum. Refusals were measured daily to estimate mean DM intake. Refusals were always less than 2% of the total administered. The ingredients and the determined nutrient content of the three feeding regimens are shown in Table 1. The TMR were analyzed for chemical composition, according to Association of Official Analytical Chemists AOAC (2000) as follows: fat was determined using the Soxhlet extraction procedure (Method 991.36), crude protein was measured by the Kjeldahl nitrogen × 6.25 method (Method 968.06), and starch was quantified using the amyloglucosidase–α-amylase method (Method 996.11). The NDF was determined with an ANKOM fiber analyzer (Van Soest et al., 1991) and corrected for residual acid-insoluble ash. The concentration of net energy for lactation (NE_L_) per unit of DM intake was estimated using the equation proposed by the NRC (2007). Oxyphenol® was administered to cows in the ADD group once daily throughout the entire dry period and continued until 60 days postpartum, at a dosage of 35 g per head per day. The supplement was orally administered to each head of the ADD group in the self-locking head gate in the feeding front when TMR was unloaded. It was mixed with water to obtain a cream, which was then administered directly in the mouth using a large syringe, as described by Maggiolino et al., 2019. The chemical composition and antioxidant activity of Oxyphenol® are reported in Table 2, as determined according to the methods described by Maggiolino et al., 2020.

**Table 1:** Feed administered (ingredients and nutritional components) to dairy cows during the experimental period.

Ingredients (% of DM)	Days relative to calving		
	From -60 to -21	From -21 to +7	From + 7 to +60
Grass hay	32	18	-
Alfalfa hay	8	-	-
Triticale silage	25	30	30
Chopped cereal straw	15	8	-
Dried sugar beet pulp	9	6	5
Wheat bran	8	2	-
Mineral–vitamin premix (no anionics)	2	2.8	1.8
Live yeast/buffer	1	0.2	-
Sulla hay	-	8	12
Ground corn	-	9	21
Ground barley	-	6	10
Soybean meal	-	10	10
Grass hay	-	-	5
Rapeseed meal (canola meal)	-	-	4
Rumen‑protected fat (Ca salts)	-	-	1.2

Nutritional components (% of DM)			
Crude protein	13.2	15.8	17
Neutral detergent fiber	44	36	32
Starch	11	19	27
Sugars	6.7	6.6	5
Fat	2.8	3.3	5.5
Net energy for lactation, MJ/kg DM	5.5	6.1	6.9
Calcium	0.60	0.82	0.90
Phosphorus	0.35	0.40	0.45
Magnesium	0.35	0.45	0.40

**Table 2:** Composition and antioxidant activity of *Pinus taeda* hydrolyzed lignin extract (Oxyphenol®) fed to dairy cow of treated group (ADD) for 120 days.

Components (g/kg)	
Vanillin	264
Eriodictyol	34
Quercetin	27
Isorhamnetin	16
Rosmarinic acid	14
Quercetin rhamnoside	139
Methyl gallate rutinoside	423
Epigallocatechin-3-methylgallate	15
Ferulic acid derivatives	67

Antioxidant activity (μmol TE^1^ g−1 DW^2^)	
TEAC^3^	23.9
ORAC^4^	122.44

1 TE = Trolox equivalents.

2 DW = dry weight.

3 TEAC = Trolox equivalent antioxidant capacity.

4 ORAC = oxygen radical absorbance capacity.

### 2.2 Blood sampling and analysis

Blood samples were collected from the coccygeal vein into sterile Vacutainer tubes (Becton Dickinson and Co., Franklin Lakes, New Jersey, United States) after the morning milking (around 7:00 AM), at several time points: at the beginning of the dry period (T-60), 30 days into the dry period (T-30), one week before calving (T-7), three days before calving (T-3), and on the day of calving (T0). *Postpartum* sampling was performed at 3 days (T+3) and 7 days (T+7) post calving, and subsequently every 30 days during lactation (T+30, T+60). At each experimental time point three 9-mL EDTA and three 9-mL clot activator tubes were collected from each cow. Blood samples were centrifuged (1,500 × g for 10 min) to separate serum and plasma, which were then aliquoted and stored at –20°C until subsequent analyses.

#### 2.2.1 Blood biochemical profile

Serum biochemical profiles were assessed using an automated clinical chemistry analyzer (CS-300B; Dirui, Changchun, China) (Forte et al., 2025). The following variables were determined: alanine aminotransferase (ALT), aspartate aminotransferase (AST), alkaline phosphatase (ALP), glucose, urea, uric acid, creatinine, total protein (TP), albumin (Alb), cholesterol, triglycerides, non-esterified fatty acids (NEFA), calcium, phosphorus, and magnesium, employing commercial kits (Gesan Production Kit, Campobello di Mazara, Trapani, Italy). Globulin concentrations were calculated as the difference between TP and Alb values. Prior to each analytical run, the analyzer was calibrated with standard solutions supplied with the assay kits (Seracal, Gesan Production Kit, Campobello di Mazara, Trapani, Italy). Accuracy of the measurements was then checked using two control bovine sera (Seracontrol N and Seracontrol P, Gesan Production Kit, Campobello di Mazara, Trapani, Italy). Results were considered acceptable when the observed values differed by less than 3.00% from the reference values provided by the manufacturer (Maggiolino et al., 2025).

#### 2.2.2 Oxidative profile of plasma

Plasma samples (0.5 mL) were diluted in 15 mL of deionized distilled water (DDW) within 50-mL test tubes and thoroughly homogenized. From each homogenate, 1 mL was transferred into a glass tube for the determination of thiobarbituric acid reactive substances (TBARS) (Forte et al., 2025). To this aliquot, 0.05 mL of butylated hydroxytoluene (7.2% in ethanol) and 1.95 mL of a thiobarbituric acid (TBA)/trichloroacetic acid (TCA)/HCl mixture (0.375% TBA, 15% TCA, 0.25 N HCl) were added. The mixture was vortexed and subsequently incubated at 90°C for 15 min in a thermostatic water bath. Following incubation, samples were cooled to room temperature (15–30°C) and centrifuged at 2000 × g for 15 min. The absorbance of the resulting supernatant was then recorded at 531 nm, using as blank a solution containing 2 mL of TBA/TCA/HCl mixture and 1 mL of DDW. TBARS concentrations were quantified against a standard calibration curve prepared with 1,1,3,3-tetramethoxypropane, and lipid peroxidation was expressed as milligrams of malondialdehyde per milliliter of plasma.

Plasma samples (0.5 mL) were diluted in 20 mL of 0.15 M KCl and incubated for 2 min. Two aliquots of the homogenate (50 μL each) were then mixed with 1 mL of 10% trichloroacetic acid (TCA) and centrifuged at 1200 × g for 3 min at 4°C to prepare for protein carbonyl quantification (Maggiolino et al., 2021). One aliquot, serving as the reference, was treated with 1 mL of 2 M HCl, whereas the other was reacted with 1 mL of 2 M HCl containing 10 mM 2,4-dinitrophenylhydrazine (DNPH). Both tubes were incubated for 1 h at room temperature (15–30°C), with gentle mixing every 20 min. After incubation, 1 mL of 10% TCA was added to each sample, followed by vortexing (30 s) and centrifugation at 1200 × g for 3 min at 4°C. This washing step was repeated three times, carefully discarding the supernatant each time. The resulting pellets were subsequently washed with 1 mL of ethanol:ethyl acetate (1:1, v/v), vortexed, and centrifuged under the same conditions three times before removing the supernatant. Finally, pellets were resuspended in 1 mL of 20 mM sodium phosphate buffer containing 6 M guanidine hydrochloride, shaken, and centrifuged at 1200 × g for 3 min at 4°C. The absorbance of the DNPH-derivatized samples was read at 360 nm using a Beckman Coulter DU800 spectrophotometer (Beckman Instruments Inc., Brea, CA, USA). Protein carbonyl levels were expressed as nanomoles of carbonyl groups per milligram of protein. Protein concentration was determined by the Biuret method.

The ferric reducing ability of plasma (FRAP) assay was used to evaluate the total antioxidant capacity, following the procedure of Benzie and Strain (1996) with minor adjustments. Briefly, 3 mL of freshly prepared FRAP working solution was obtained by combining 1 mL of 10 mM TPTZ dissolved in 40 mM HCl, 1 mL of 20 mM FeCl₃ in distilled water, and 10 mL of 300 mM acetate buffer (pH 3.6). After addition of 100 μL of plasma sample or supernatant, the mixture was incubated at 37°C for 40 min. The absorbance was then measured at 593 nm, and results were expressed as μmol Trolox equivalents per ml.

#### 2.2.3 Antioxidant enzyme activity of plasma

Superoxide dismutase (SOD; EC 1.15.1.1) activity was determined following the principles outlined by Misra (2018). The method relies on the ability of SOD to inhibit the autoxidation of epinephrine, with the inhibitory effect quantified spectrophotometrically at 480 nm (Dinardo et al., 2021). To prevent spurious oxidation caused by trace metal contaminants in the reagents, the assay buffer was supplemented with 10–4 M EDTA to chelate divalent cations. Activity of SOD was expressed as units per milliliter (U/mL), where one unit corresponds to the amount of enzyme that produces 50% inhibition of epinephrine autoxidation. Glutathione peroxidase (GSPx; EC 1.11.1.9) activity was assessed following the procedure described by Flohé & Günzler (1984) and further detailed by Dinardo et al. (2022). The assay evaluates the GS-Px-dependent oxidation of reduced glutathione (GSH) by tert-butyl hydroperoxide. To maintain GSH at a constant level throughout the reaction, exogenous glutathione reductase and NADPH were added, enabling the rapid recycling of oxidized glutathione back to its reduced form. The consumption of NADPH was tracked by monitoring the decrease in absorbance at 340 nm, and enzymatic activity was reported as U/mL. Glutathione S-transferase (GST; EC 2.5.1.18) activity was measured using 1-chloro-2,4-dinitrobenzene (CDNB) as the substrate, according to the method of Habig & Jakoby (1981). The assay is based on the conjugation of GSH with CDNB, and the rate of product formation was determined spectrophotometrically by recording the increase in absorbance at 340 nm. The results were expressed as U/mL of enzyme activity.

#### 2.2.4 Determination of reactive oxygen species production and cell viability in peripheral blood mononuclear cell

Peripheral blood mononuclear cells (PBMC) were separated from whole blood following a density gradient centrifugation procedure, as described by Maggiolino et al., 2024. 20 milliliters of whole blood were diluted 1:1 with cold phosphate-buffered saline (PBS) and gently layered onto 10 mL of Histopaque®-1077 (Sigma–Aldrich, Milan, Italy). Samples were centrifuged at 400 × g for 30 min at 20°C, and the mononuclear cell layer was carefully collected. The recovered PBMC were washed twice with PBS, and the final pellet was resuspended in calcium- and magnesium-free Iscove’s Modified Dulbecco’s Medium (IMDM; Sigma–Aldrich). Cell concentration was determined using a Bürker hemocytometer, while cell viability was assessed by Trypan Blue exclusion (Sigma–Aldrich, Milan, Italy). For stimulation assays, PBMC suspensions (2 × 10⁵ cells/well in 100 µL) were plated in 96-well U-bottom plates with serum-free IMDM and treated either with concanavalin A (ConA; 5 µg/mL final concentration) or hydrogen peroxide (H₂O₂; 1 mM final concentration). Untreated cells served as negative controls. After 20 h of incubation at 37°C in 5% CO₂, plates were centrifuged at 400 × g for 10 min at 20°C. The culture supernatants were further clarified at 10,000 × g, and aliquots were stored at −80°C until further analysis. Intracellular ROS generation was quantified using the fluorescent probe 2’,7’-dichlorodihydrofluorescein diacetate (DCFH-DA), as previously reported (Maggiolino et al., 2024). PBMC (2 × 10⁵ cells/well in 96-well plates) were incubated with 10 µM DCFH-DA in phenol red–free IMDM for 30 min at 37°C. Subsequently, cells were exposed for 1 h and 30 min to either ConA (5 µg/mL) or H₂O₂ (1 mM). After treatment, the medium was discarded, and cells were rinsed twice with PBS. The fluorescence intensity was measured in phenol red–free IMDM using a spectrofluorometer (excitation 485 nm, emission 525 nm). Control wells followed the same protocol but without oxidant exposure. Production of ROS was expressed as the percentage change in fluorescence relative to the untreated control. Extracellular ROS levels in PBMC supernatants were assessed using the OxiSelect™ In Vitro ROS Assay Kit (Green Fluorescence; Cell Biolabs Inc., San Diego, CA, USA), following the manufacturer’s protocol. The assay is based on the oxidation of DCFH to the fluorescent product DCF by reactive species. Fluorescence was measured at 480 nm excitation and 530 nm emission using a VersaMax Microplate Reader (Molecular Devices, Sunnyvale, CA, USA). Blank values were subtracted from all measurements to remove background signal, and the resulting fluorescence intensity, expressed in arbitrary units, was proportional to the total ROS content of each sample. Cell viability and cytotoxicity of PBMC from the different experimental groups were evaluated using the MTT [3-(4,5-dimethylthiazol-2-yl)-2,5-diphenyltetrazolium bromide] assay, as previously described by Di Bari et al. (2014). This method relies on the ability of mitochondrial succinate dehydrogenase in metabolically active cells to reduce MTT to insoluble formazan crystals, which are then solubilized and quantified spectrophotometrically. Briefly, after removing the culture medium, cells were washed with PBS and incubated for 2 h at 37°C and 5% CO₂ with MTT solution (0.5 mg/mL). Plates were centrifuged, the medium discarded, and the formazan crystals dissolved in absolute ethanol. Absorbance was measured at 560 nm and 690 nm using a VersaMax Microplate Reader (Molecular Devices). Cell viability was expressed as the percentage of the absorbance of treated samples relative to untreated controls (set as 100%).

### 2.3 Colostrum and milk sampling and analysis

The first colostrum sample was collected within 2 h after calving from each cow enrolled in the trial. Thereafter, transition milk was collected once daily in the early morning from day 2 to day 5 postpartum. Colostrum/transition milk quality was assessed using Brix refractometry. Milk samples were collected up to 60 days of lactation at 15-day intervals. Milking was performed in a conventional parlor twice daily, at 06:00 and 17:00 h. At the end of each milking, 100 mL of milk per cow was collected, providing a sample representative of the full yield. Immediately after collection, samples were stored at 4°C, transported to the laboratory, and combined in proportion to the morning and evening yields. The pooled milk was then analyzed within 2 h (below).

#### 2.3.1 Chemical composition of colostrum and milk

Moisture, protein, and ash contents of colostrum were determined following the International Organization for Standardization (ISO) protocols (ISO, 1973, 1978, 1998), whereas lipid content was analyzed according to the AOCS Am 5-04 official method (Association of Official Analytical Chemists AOAC, 2000). Moisture was quantified by drying samples in an oven at 105°C for 24 h (Memmert UFP 600, Schwabach, Germany), with results expressed as the weight loss between the initial and final measurements. Protein concentration was assessed using the Kjeldahl procedure: samples were digested with sulfuric acid in the presence of copper sulfate catalyst (Kjeldatherm KB system, Gerhardt, Bonn, Germany), generating ammonium sulfate, which was then distilled under alkaline conditions using a Vapodest 50 Carrousel unit (Gerhardt, Bonn, Germany). Total nitrogen was measured and converted to crude protein by applying the factor 6.25. Ash determination was performed by incinerating the material at 550°C for 4–5 h in a muffle furnace (Carbolite RWF 1200, Hope Valley, UK), with mineral residue expressed relative to the original sample weight. For lipid content assessment, samples were subjected to acid hydrolysis followed by lipid extraction with petroleum ether at 90°C for 60 min using an AnkomHCI Hydrolysis System (Macedon, NY, USA). The extracted lipid fraction was then quantified gravimetrically.

Near-infrared spectroscopy was used to determine fat, protein, casein, lactose, short-chain fatty acids (SFA), unsaturated fatty acids (UFA), monounsaturated fatty acids (MUFA), and polyunsaturated fatty acids (PUFA) in milk (ISO, 2013). Milk coagulation properties, including clotting time, curd-firming rate, and curd firmness, were assessed with a Formagraph (Foss Electric A/S, Hillerød, Denmark) following the protocol described by (Dadousis et al. (2016).

#### 2.3.2 Oxidative profile of colostrum and milk

Lipid oxidation was assessed using the thiobarbituric acid reactive substances (TBARS) assay, as reported by Oancea et al., 2022. Briefly, 0.5 mL of milk was mixed with 1 mL of 20% trichloroacetic acid (TCA) and incubated for 15 min at room temperature. The samples were then centrifuged at 3000 × g for 10 min, and 1 mL of the supernatant was transferred to a clean tube. To this, 0.6 mL of 0.8% thiobarbituric acid (TBA) solution was added. The mixture was incubated at 80°C for 90 min and subsequently cooled to room temperature (15–30°C). Absorbance was measured at three different wavelengths to identify degradation products specific to milk oxidation: 450 nm (saturated aldehydes), 495 nm (dienals), and 532 nm (malondialdehyde), using a Shimadzu IRTracer-100 spectrophotometer. A blank solution containing TBA/TCA/HCl and distilled water was used as reference. Malondialdehyde was quantified using a standard calibration curve prepared with 1,1,3,3-tetramethoxypropane and expressed in µg/l of milk.

Protein oxidation was determined by measuring protein carbonyl content. Two aliquots of milk (50 μL each) were treated with 1 mL of 10% TCA, followed by centrifugation at 1200 × g for 3 min at 4°C. One aliquot served as a blank and was treated with 1 mL of 2 M HCl, while the second was incubated with 1 mL of 2 M HCl containing 10 mM 2,4-dinitrophenylhydrazine (DNPH). Samples were left at room temperature (15–30°C) for 1 h with intermittent shaking every 20 min. Subsequently, 1 mL of 10% TCA was added, and the mixtures were vortexed (30 s) and centrifuged three times at 1200 × g for 3 min at 4°C. The pellets were washed three times with 1 mL ethanol:ethyl acetate (1:1, v/v) under the same centrifugation conditions. Pellets were finally solubilized in 1 mL of 20 mM sodium phosphate buffer containing 6 M guanidine hydrochloride. Carbonyl concentration was determined spectrophotometrically at 360 nm on DNPH-treated samples using a Beckman Coulter DU800 spectrophotometer and expressed as nmol carbonyls per mg of protein. Protein concentration was measured using the Biuret method, following the procedure of Tokur and Korkmaz (2007).

Antioxidant capacity of milk was evaluated by the FRAP assay. For each sample, 300 μL of milk was added to 4.5 mL of freshly prepared FRAP reagent. After vortex mixing, samples were incubated at 37°C for 10 min and vortexed again. Subsequently, 4 mL of the reaction mixture was withdrawn with a syringe, filtered through a 0.45 μm pore filter, and the eluate was collected. From this, 250 μL per sample was pipetted in triplicate into the wells of a 96-well microplate. Absorbance was read immediately at 595 nm using a microplate reader. A blank was prepared by substituting milk with 300 μL of distilled water while keeping the reagent volume constant. Final values were corrected by subtracting the blank absorbance. Antioxidant capacity was expressed as nmol ascorbic acid equivalents per mL of milk, using a calibration curve generated with ascorbic acid standards.

### 2.4 Statistical analysis

The freeware software Lenth, R. V. (2006-9) was used to determine the minimum sample size. (Retrieved 15/01/2023, from http://www.stat.uiowa.edu/∼rlenth/Power.). The experimental design considered the 2 groups and the 9 times, and for sample calculation, the value of α was set to 0.05 and β to 0.20, for a power of 0.80. The expected difference was set at 0.17. The plasma TBARS level and its standard deviation (0.14) were the outcome considered for sample size calculation. The sample size obtained was 10 cows.

Prior to statistical analysis, data were checked for normality and homogeneity of variances using the Shapiro–Wilk test and inspection of residual plots. Since the same cows were sampled repeatedly over time, data were reanalysed using a mixed model for repeated measures with the MIXED procedure of SAS software (version 9.3; SAS Institute Inc., Cary, NC, USA; SAS, 2018). For blood variables, the model included treatment, sampling day, and their interaction as fixed effects, with nine sampling days considered: T-60, T-30, T-7, T-3, T0, T+3, T+7, T+30, and T+60. Cow nested within treatment was considered the experimental subject for repeated measures. Calving month was included as an adjustment factor. For colostrum variables, five sampling days were considered, whereas for milk variables four sampling days were considered. Several covariance structures, including compound symmetry, autoregressive [AR(1)], heterogeneous autoregressive [ARH(1)], Toeplitz, and unstructured covariance matrices, were compared. The covariance structure providing the lowest Akaike information criterion was retained for each variable. The model employed was structured as follows:

$$y_{\text{ijkl}}=\mu +T_{i}+D_{j}+\left(T\times D\right)i_{\text{jk}}+C_{k}\left(T_{i}\right)+M_{l}+\varepsilon_{\text{ijkl}},$$

where y_ijkl_ is the dependent variable; μ is the overall mean; T_i_ is the fixed effect of the treatment, D_j_ is the fixed effect of sampling day, (T × D)_ij_ is the treatment × sampling day, C_k_(T_i_) is the effect of cow nested within treatment, M_l_ is the effect of calving month, and ε_ijkl_ is the residual error. When significant effects were detected, least squares means were compared using Bonferroni adjustment for treatment comparisons within sampling day and Tukey adjustment for comparisons among sampling days. Cell viability data were analysed separately for each in vitro stimulation condition, namely ConA and H₂O₂. Since PBMC viability was assessed repeatedly in cells obtained from the same cows at different sampling days, data were analysed using a repeated-measures mixed model. The model included treatment, sampling day, and their interaction as fixed effects, with cow nested within treatment as the repeated subject and calving month as an adjustment factor. When significant effects were detected, least squares means were compared using Bonferroni adjustment for treatment comparisons within each sampling day. Data are reported as least squares means with pooled standard error of the mean. Statistical significance was declared at *P* < 0.05, whereas 0.05 ≤ *P* < 0.10 was considered a tendency.

## 3 Results

Table 3 presents the fixed effects of treatment, sampling day, and their interaction on the blood biochemical profile of the cows. Sampling day affected total protein, globulin, cholesterol, triglycerides, NEFA, phosphorus, and AST (*P* < 0.01), as well as glucose (*P* < 0.05). Treatment, on the other hand, affected ALT (*P* < 0.05) and ALP (*P* < 0.01). Specifically, ALT showed higher concentrations in CON group, compared to ADD (*P* < 0.05), as well as ALP (*P* < 0.01).

**Table 3:** Blood biochemical profile of dairy cows fed 35g/day/head with (ADD; n = 10) or without (CON; n = 10) Oxyphenol® (*Pinus taeda* hydrolyzed lignin) from the beginning of the dry period through 60 days in lactation.

Parameter	Group^1^	Days relative to calving									SEM^2^	Analysis of variance^3^		
		-60	-30	-7	-3	0	+3	+7	+30	+60		T	D	T × D
Total protein (g/dl)	CON	6.72^CB^	7.01	6.35^C^	6.69^CB^	6.75^CB^	7.31	6.81	7.61^AB^	7.76^A^	0.27	0.769	<0.001	0.772
	ADD	7.04^AB^	7.38	6.61^B^	6.57^B^	6.64^B^	6.95	7.11	7.72^A^	7.32^AB^				
Albumin (g/dl)	CON	4.21	3.87	3.78	3.78	3.90	4.19	3.71	3.90	4.10	0.12	0.318	0.283	0.057
	ADD	3.64	4.07	3.97	3.79	3.75	3.84	3.86	3.93	4.05				
Globulin (g/dl)	CON	2.57^B^	3.15	2.63^B^	2.76	2.88	3.04	3.20	3.69^A^	3.70^A^	0.28	0.398	0.004	0.812
	ADD	3.32	3.38	2.69^B^	2.78^ABb^	3.0^B^	3.11	3.22	3.82^Aa^	3.28				
Urea (mg/dl)	CON	28.97	25.54	30.57	32.75	29.62	26.00	26.86	27.10	28.70	3.05	0.124	0.111	0.769
	ADD	29.02	29.17	38.37	31.30	32.25	33.42	28.93	26.48	25.64				
Uric acid (mg/dl)	CON	1.45	1.32	1.29	1.20	1.22	1.42	1.63	1.55	1.67	0.19	0.063	0.375	0.990
	ADD	1.28	1.10	1.15	1.16	1.13	1.35	1.25	1.48	1.36				
Creatinine (mg/dl)	CON	1.51	1.61	1.57	1.56	1.61	1.59	1.41	1.36	1.35	0.10	0.549	0.385	0.813
	ADD	1.31	1.50	1.54	1.57	1.65	1.52	1.42	1.29	1.27				
Cholesterol (mg/dl)	CON	119.62^BC^	93.14^BC^	75.97^D^	76.02^D^	67.31^D^	82.22^D^	88.66^CD^	150.20^AB^	174.66^A^	9.95	0.142	<0.001	0.838
	ADD	120.95^B^	103.24^BC^	73.53^CD^	72.67^CD^	58.82^D^	65.17^D^	80.35^CD^	127.42^B^	165.28^A^				
Triglycerides (mg/dl)	CON	30.97	34.70^AB^	35.17^AB^	36.80^A^	30.60	23.20	15.82^C^	21.72	19.72^BC^	4.59	0.079	0.002	0.816
	ADD	25.92	27.48^A^	28.64^A^	29.90^A^	21.47	18.64^B^	20.33	18.17^B^	22.46				
Non-esterified fatty acids (mmol/l)	CON	0.40^A^	0.37^A^	0.45^a^	0.60	0.83^Bb^	0.63	0.44^a^	0.24^A^	0.36^A^	0.09	0.181	<0.001	0.643
	ADD	0.37^B^	0.31^B^	0.25^B^	0.38^B^	0.71^A^	0.56^B^	0.34^B^	0.13^C^	0.21^BC^				
Glucose (mg/dl)	CON	57.60^a^	56.44	56.71	56.28	53.11	41.60^b^	43.14^b^	48.66	48.60	6.20	0.648	0.013	0.933
	ADD	59.67^A^	54.88	51.46	54.45	54.95	50.95	42.75^B^	50.51	51.33				
Magnesium (mg/dl)	CON	2.56	2.83	2.57	2.85	2.62	3.23	2.50	3.29	3.46	0.30	0.582	0.057	0.919
	ADD	2.52	2.38	2.63	2.73	3.07	2.83	2.63	3.18	3.22				
Phosphorus (mg/dl)	CON	7.06	7.37	6.74	6.91	6.90	6.48^B^	7.31	8.27^A^	8.21^A^	0.44	0.315	0.003	0.710
	ADD	7.12	7.28	6.80	6.64	6.14^B^	6.52	7.97^A^	7.13	7.78				
Calcium (mg/dl)	CON	8.50	8.89	8.68	8.79	8.60	7.82	8.50	8.62	8.36	0.33	0.267	0.227	0.806
	ADD	8.67	8.93	8.52	8.42	7.75	8.25	8.25	8.40	7.97				
ALT^4^ (U/l)	CON	17.31	17.92	19.71	17.64	19.44	26.04	16.42	17.04	21.48	3.12	0.025	0.581	0.593
	ADD	15.70	15.66	16.28	14.60	14.85	14.22	13.17	19.48	20.15				
AST^5^ (U/l)	CON	35.75	29.70^B^	38.11	34.31	40.68	42.95	41.93	53.52	60.86^A^	7.81	0.434	0.003	0.997
	ADD	28.95	35.11	35.37	29.30	34.67	40.84	35.42	55.42	55.97				
ALP^6^ (U/l)	CON	185.31	162.58	189.60	181.60	207.17	176.84	189.86	162.40	174.42	35.38	0.002	0.998	0.993
	ADD	114.07	134.06	125.06	114.67	131.20	130.46	133.97	140.40	155.53				
Bilirubin (mg/dl)	CON	0.49	0.46	0.43	0.48	0.55	0.55	0.44	0.42	0.41	0.05	0.458	0.050	0.992
	ADD	0.49	0.42	0.41	0.45	0.59	0.56	0.37	0.39	0.39				

A, B, C, D – Means with different superscripts differ significantly (*P* < 0.01) due to sampling day.

a, b – Means with different superscripts differ significantly (*P* < 0.05) due to sampling day.

1 CON = control treatment, ADD = Oxyphenol® treatment (*Pinus taeda* hydrolyzed lignin).

2 SEM = standard error of the means.

3 T = treatment, D = sampling day, T × D = binary interaction between treatment and sampling day.

4 ALT = alanine aminotransferase.

5 AST = aspartate aminotransferase.

6 ALP = alkaline phosphatase.

Table 4 reports the fixed effects of treatment, sampling day, and their interaction on the oxidative profile and antioxidant enzyme activity of plasma. Treatment affected TBARS levels, SOD (*P* < 0.01), and GSPx (*P* < 0.05), whereas sampling day affected SOD (*P* < 0.01) and TBARS (*P* < 0.05). In CON group TBARS concentrations were higher than those in ADD group (*P* < 0.01)In ADD group, SOD activity was lower than in the CON group (*P* < 0.01). The GSPx activity was also higher in the CON treatment than in ADD (*P* < 0.05).

**Table 4:** Oxidative profile and antioxidant enzyme activity of plasma of dairy cows fed 35g/day/head with (ADD; n = 10) or without (CON; n = 10) Oxyphenol® (*Pinus taeda* hydrolyzed lignin) from the beginning of the dry period through 60 days into lactation.

Parameter	Group^1^	Days relative to calving									SEM^2^	Analysis of variance^3^		
		-60	-30	-7	-3	0	+3	+7	+30	+60		T	D	T × D
TBARS^4^ (mg malondialdehyde/ml plasma)	CON	1.40^b^	1.38	1.32	1.72	2.31	3.84^a^	3.36	3.19	2.78	0.41	0.002	0.031	0.197
	ADD	1.62	1.54	1.33	2.19	1.88	1.94	1.98	1.49	1.42				
Protein carbonyls (nm carbonyl groups/mg protein)	CON	77.20	78.06	72.70	69.09	69.43	73.01	74.11	83.08	80.57	6.31	0.082	0.203	0.882
	ADD	80.11	71.63	71.05	64.88	68.24	61.56	66.67	72.90	76.32				
FRAP^5^ (μmol Trolox equivalents/ml)	CON	92.51	86.41	93.57	89.35	80.18	99.06	96.57	99.94	110.18	6.95	0.156	0.090	0.933
	ADD	88.60	84.23	86.11	85.99	86.89	102.17	105.19	115.96	118.29				
Superoxide dismutase (U/ml)	CON	26.65^C^	27.86	37.00^B^	39.64^B^	46.00^A^	48.60^A^	41.45	36.93^B^	28.28	3.88	0.005	0.001	0.141
	ADD	29.90	29.63	32.58^A^	30.25	28.30	31.50^A^	30.08	28.50	21.06^B^				
Glutathione peroxidase (U/ml)	CON	3.76	2.99	2.96	3.47	4.23	4.78	3.70	4.38	3.98	0.63	0.022	0.113	0.229
	ADD	3.75	3.14	3.23	3.32	3.88	3.70	3.28	3.25	3.02				
Glutathione S-transferase (U/ml)	CON	0.24	0.33	0.38	0.33	0.45	0.35	0.39	0.35	0.23	0.04	0.054	0.321	0.515
	ADD	0.26	0.36	0.24	0.21	0.28	0.26	0.24	0.22	0.21				

A, B, C – Means with different superscripts differ significantly (*P* < 0.01) due to sampling day.

a, b – Means with different superscripts differ significantly (*P* < 0.05) due to sampling day.

1 CON = control treatment, ADD = Oxyphenol® treatment (*Pinus taeda* hydrolyzed lignin).

2 SEM = standard error of the means.

3 T = treatment, D = sampling day, T × D = binary interaction between treatment and sampling day.

4 TBARS = Thiobarbituric acid reactive substances.

5 FRAP = Ferric reducing ability of plasma.

**Fig. 1:**
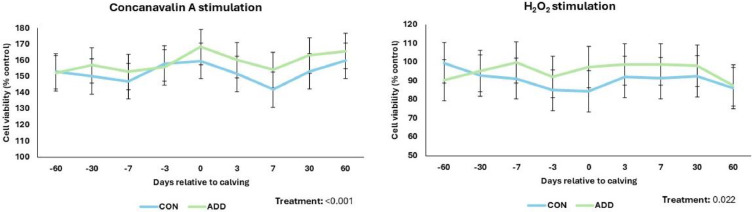
Cell viability and proliferation of peripheral blood mononuclear cells (PBMC) treated in vitro with concanavalin A and hydrogen peroxide of dairy cows fed with (ADD) or without (CON) Oxyphenol® (*Pinus taeda* hydrolyzed lignin) from the beginning of the dry period through 60 days into lactation.

Fig. 1 shows the effect of treatment on the cell viability and proliferation of PBMC treated in vitro with ConA and H_2_O_2_. Treatment affected cell viability after stimulation with both ConA (*P* < 0.01) and H_2_O_2_ (*P* < 0.05). Compared to CON group, ADD group showed an higher viability after ConA stimulation (*P* < 0.01), and also after H_2_O_2_ stimulation (*P* < 0.05).

**Fig. 2:**
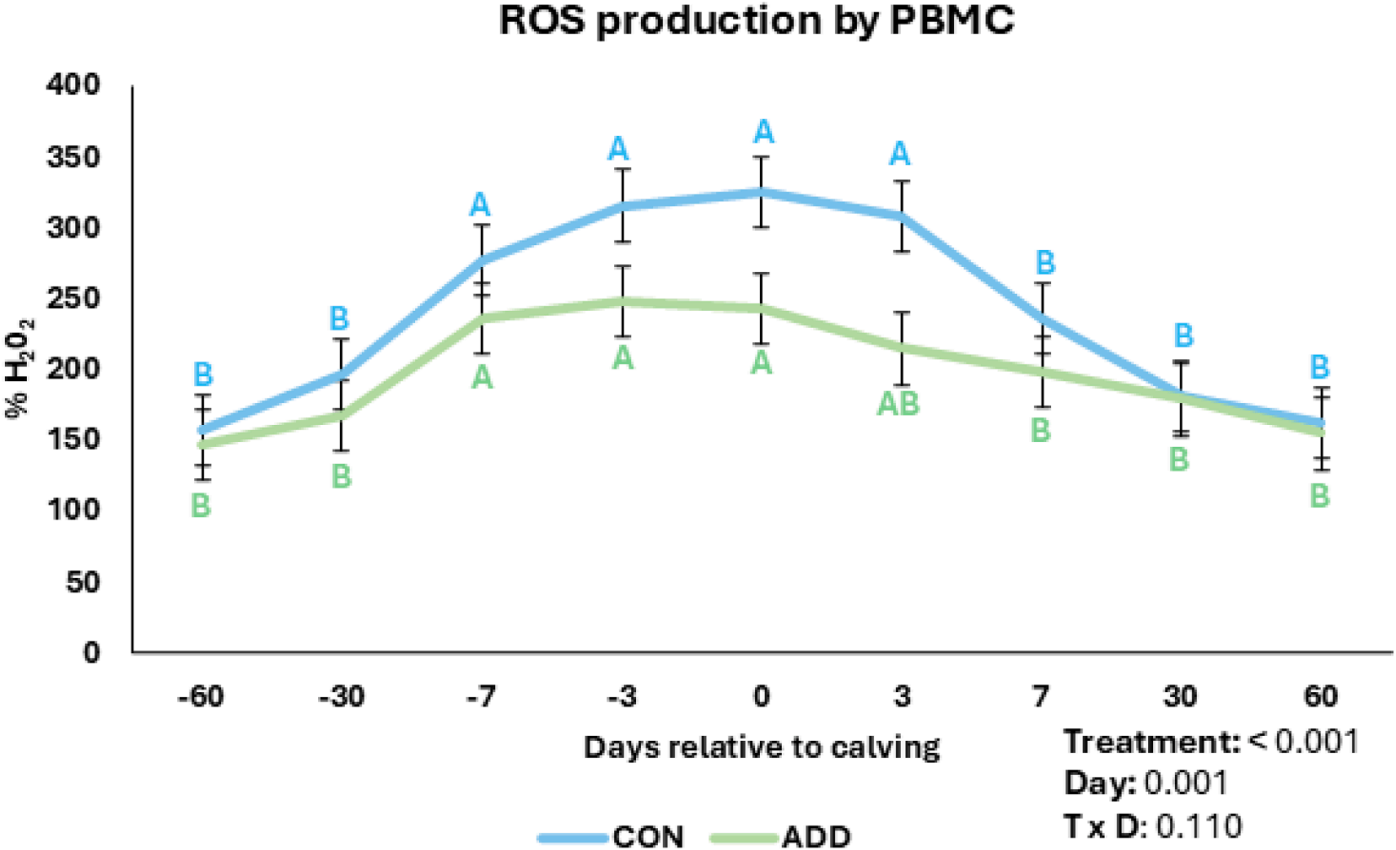
Reactive oxygen species (ROS) production by peripheral blood mononuclear cells (PBMC) of dairy cows fed with (ADD) or without (CON) Oxyphenol® (*Pinus taeda* hydrolyzed lignin) from the beginning of the dry period through 60 days into lactation.

The production of ROS by PBMC is reported in Fig. 2. Both treatment and sampling day affected ROS levels (*P* < 0.01), and ADD group showed lower levels of ROS compared to CON group (*P* < 0.01).

The fixed effects of treatment, sampling day, and their interaction on milk production parameters (Table 5) and coagulation properties (Table 6) were evaluated, and none of these parameters were influenced by any of the fixed effects (*P* > 0.05), except for milk yield which was affected by sampling day (*P* < 0.01).

**Table 5:** Milk yield and chemical composition of milk from dairy cows fed with 35g/day/head with (ADD; n = 10) or without (CON; n = 10)) Oxyphenol® (Pinus taeda hydrolyzed lignin) from the beginning of the dry period through 60 days into lactation.

Parameter	Group^1^	Days *postpartum*				SEM^2^	Analysis of variance^3^		
		15	30	45	60		T	D	T × D
Milk yield	CON	29.83^C^	35.72^Bb^	38.56^Ba^	41.10^A^	7.24	0.392	<0.001	0.283
	ADD	30.22^C^	36.48^Bb^	39.45^Ba^	42.23^A^				
Fat (g/100g)	CON	4.21	3.66	3.69	3.30	0.63	0.697	0.626	0.296
	ADD	4.81	3.82	4.87	4.53				
Protein (g/100g)	CON	3.58	3.47	3.36	3.59	0.08	0.235	0.109	0.296
	ADD	3.71	3.51	3.55	3.49				
Casein (g/100g)	CON	2.86	2.75	2.68	2.84	0.06	0.364	0.078	0.493
	ADD	2.94	2.77	2.80	2.77				
Whey proteins (g/100g)	CON	0.71	0.72	0.67	0.75	0.02	0.131	0.605	0.152
	ADD	0.77	0.73	0.74	0.72				
Lactose (g/100g)	CON	4.74	4.82	4.70	4.86	0.07	0.285	0.680	0.305
	ADD	4.82	4.90	4.86	4.75				
Urea (mg/dl)	CON	28.35	24.73	26.23	32.33	2.80	0.552	0.628	0.324
	ADD	28.90	28.08	31.67	27.71				
BHB (mM)	CON	0.01	0.01	0.01	0.01	0.00	0.309	0.951	0.941
	ADD	0.02	0.02	0.01	0.02				
pH	CON	6.55	6.61	6.58	6.62	0.03	0.522	0.644	0.496
	ADD	6.66	6.68	6.69	6.63				
Fat-free dry matter (g/100g)	CON	9.06	9.01	8.75	9.16	0.14	0.214	0.421	0.203
	ADD	9.27	9.15	9.12	8.94				
Citric acid	CON	0.10	0.09	0.10	0.10	0.00	0.441	0.788	0.711
	ADD	0.08	0.10	0.09	0.10				
Somatic cell score	CON	3.26	3.97	4.00	4.08	0.65	0.828	0.752	0.809
	ADD	3.86	3.87	3.84	3.85				
Total bacterial load (UFC/ml)	CON	11665.81	12245.8	8841.03	16961.78	0.70	0.293	0.198	0.944
	ADD	12311.37	13967.93	9944.03	14053.61				

A, B,C – Means with different superscripts differ significantly (*P* < 0.01) due to sampling day.

a, b – Means with different superscripts differ significantly (*P* < 0.05) due to sampling day.

1 CON = control treatment, ADD = Oxyphenol® treatment (*Pinus taeda* hydrolyzed lignin).

2 SEM = standard error of the means.

3 T = treatment, D = sampling day, T × D = binary interaction between treatment and sampling day.

**Table 6:** Coagulation properties of milk from dairy cows fed with 35g/day/head with (ADD; n = 10) or without (CON; n = 10) Oxyphenol® (*Pinus taeda* hydrolyzed lignin) from the beginning of the dry period through 60 days into lactation.

Parameter	Group^1^	Days *postpartum*				SEM^2^	Analysis of variance^3^		
		15	30	45	60		T	D	T × D
a30^4^ (mm)	CON	26.25	26.43	26.37	25.96	2.89	0.366	0.398	0.345
	ADD	26.35	26.14	26.50	26.92				
k20^5^ (min)	CON	10.18	11.12	10.75	10.89	0.66	0.577	0.511	0.329
	ADD	11.64	11.57	11.21	11.42				
RCT^6^ (min)	CON	14.12	14.18	14.81	15.52	2.39	0.341	0.211	0.298
	ADD	14.50	15.47	15.78	15.00				

1 CON = control treatment, ADD = Oxyphenol® treatment (*Pinus taeda* hydrolyzed lignin).

2 SEM = standard error of the mean.

3 T = treatment, D = sampling day, T × D = binary interaction between treatment and sampling day.

4 a30 = Curd firmness 30 min after rennet addition

5 k20 = time to reach a curd firmness of 20 mm

6 RCT = rennet coagulation time.

Table 7 shows the fixed effects of treatment, sampling day and their interaction on milk oxidative profile. Treatment affected malondialdehyde and FRAP (*P* < 0.01). Malondialdehydes were higher in CON group than in the ADD one (*P* < 0.01). FRAP values were higher in ADD group than in the CON group (*P* < 0.01). The fixed effects of treatment, sampling day, and their interaction on the fatty acids profile of milk, presented in Table 8, showed no significant influence (*P* > 0.05).

**Table 7:** Oxidative profile of milk from dairy cows fed with 35g/day/head with (ADD; n = 10) or without (CON; n = 10) Oxyphenol® (*Pinus taeda* hydrolyzed lignin) from the beginning of the dry period through 60 days into lactation.

Parameter	Group^1^	Days *postpartum*				SEM^2^	Analysis of variance^3^		
		15	30	45	60		T	D	T × D
Malondialdehyde (μg/L)	CON	69.51	68.05	70.21	70.89	4.00	0.001	0.352	0.106
	ADD	63.62	61.42	59.33	57.05				
Protein carbonyls (nmol/mg protein)	CON	38.40	40.03	40.26	40.77	2.15	0.061	0.894	0.307
	ADD	39.77	37.84	38.81	37.58				
FRAP^4^ (nmol ascorbic acid eq./mL milk)	CON	176.23	170.43	167.07	159.82	20.66	0.005	0.070	0.792
	ADD	190.29	184.58	179.51	177.07				

1 CON = control treatment, ADD = Oxyphenol® treatment (*Pinus taeda* hydrolyzed lignin).

2 SEM = standard error of the mean.

3 T = treatment, D = sampling day, T × D = binary interaction between treatment and sampling day.

4 FRAP = Ferric reducing antioxidant power.

**Table 8:** Fatty acids profile of milk from dairy cows (expressed in % on milk) fed with 35g/day/head with (ADD; n = 10) or without (CON; n = 10) Oxyphenol® (*Pinus taeda* hydrolyzed lignin) from the beginning of the dry period through 60 days into lactation.

Parameter	Group^1^	Days *post-partum*				SEM^2^	Analysis of variance^3^		
		15	30	45	60		T	D	T × D
SFA (%)	CON	3.11	2.42	3.18	2.13	0.43	0.846	0.599	0.326
	ADD	2.74	2.35	2.49	3.01				
UFA (%)	CON	1.46	1.20	1.44	1.00	0.18	0.520	0.611	0.343
	ADD	1.25	1.13	1.10	1.29				
TFA^4^ (%)	CON	0.09	0.06	0.08	0.01	0.02	0.709	0.297	0.534
	ADD	0.08	0.04	0.03	0.05				
MUFA (%)	CON	1.36	1.06	1.33	0.86	0.20	0.437	0.617	0.242
	ADD	1.16	0.99	0.80	1.19				
PUFA (%)	CON	0.15	0.12	0.15	0.09	0.02	0.586	0.426	0.466
	ADD	0.14	0.11	0.11	0.12				
SCFA^5^ (%)	CON	0.68	0.53	0.70	0.43	0.09	0.639	0.542	0.312
	ADD	0.58	0.51	0.51	0.60				
MCFA^6^ (%)	CON	1.63	1.31	1.69	1.29	0.18	0.980	0.438	0.345
	ADD	1.51	1.28	1.45	1.71				
LCFA^7^ (%)	CON	1.72	1.36	1.69	1.09	0.26	0.412	0.494	0.386
	ADD	1.44	1.03	1.25	1.50				
C14:0 (%)	CON	0.43	0.33	0.45	0.33	0.05	0.862	0.481	0.353
	ADD	0.38	0.32	0.37	0.44				
C16:0 (%)	CON	1.10	0.83	1.14	0.74	0.16	0.997	0.586	0.247
	ADD	1.00	0.82	0.86	1.13				
C18:0 (%)	CON	0.46	0.36	0.42	0.26	0.07	0.839	0.400	0.583
	ADD	0.43	0.35	0.32	0.37				
C18:1 (%)	CON	1.42	1.11	1.37	0.90	0.20	0.453	0.521	0.353
	ADD	1.22	0.97	0.95	1.22				

1 CON = control treatment, ADD = Oxyphenol® treatment (*Pinus taeda* hydrolyzed lignin).

2 SEM = standard error of the mean.

3 T = treatment, D = sampling day, T × D = binary interaction between treatment and sampling day.

4 TFA = Trans-fatty acids.

5 SCFA = Short chain fatty acids.

6 MCFA = Medium chain fatty acids.

7 LCFA = Long chain fatty acids.

Table 9 shows the fixed effects of treatment, sampling day, and their interaction on the chemical composition of colostrum and transition milk. Dry matter, moisture, protein content, and Brix degrees were affected by sampling day (*P* < 0.01).

**Table 9:** Chemical composition of colostrum from dairy cows fed with 35g/day/head with (ADD; n = 10) or without (CON; n = 10) Oxyphenol® (*Pinus taeda* hydrolyzed lignin) from the beginning of the dry period through 60 days into lactation.

Parameter	Group^1^	Days *postpartum*					SEM^2^	Analysis of variance^3^		
		1	2	3	4	5		T	D	T × D
Dry matter (%)	CON	18.95^Aa^	17.12	16.36^b^	16.30^B^	15.38^B^	0.83	0.634	<0.001	0.940
	ADD	19.52^Aa^	18.02	16.34^b^	15.85^B^	15.62^B^				
Moisture (%)	CON	81.05^Bb^	82.87	83.63^a^	83.75^a^	84.61^A^	0.83	0.622	<0.001	0.945
	ADD	80.47^Bb^	81.97	83.65^a^	84.14^A^	84.37^A^				
Protein (g/100 g)	CON	8.99^A^	5.17^B^	4.53^B^	3.90^B^	4.40^B^	0.87	0.301	<0.001	0.700
	ADD	9.44^A^	7.25^a^	4.69^B^	4.50^B^	4.00^b^				
Fat (g/100 g)	CON	5.65	5.79	6.26	5.65	4.89	1.03	0.348	0.917	0.979
	ADD	4.87	4.77	5.62	4.66	5.11				
Ash (g/100 g)	CON	0.83	0.80	0.78	0.82	0.84	0.06	0.632	0.935	0.959
	ADD	0.85	0.86	0.80	0.85	0.80				
Brix (°)	CON	21.71^A^	13.87^B^	12.37^B^	12.06^B^	11.62^B^	1.28	0.230	<0.001	0.888
	ADD	22.85^A^	16.16^Ba^	13.78^B^	12.35^B^	11.42^Bb^				

A, B – Means with different superscripts differ significantly (*P* < 0.01) due to sampling day.

a, b – Means with different superscripts differ significantly (*P* < 0.05) due to sampling day.

1 CON = control treatment, ADD = Oxyphenol® treatment (*Pinus taeda* hydrolyzed lignin).

2 SEM = standard error of the mean.

3 T = treatment, D = sampling day, T × D = binary interaction between treatment and sampling day.

Table 10 reports the fixed effects of treatment, sampling day, and their interaction on the oxidative profile of colostrum. Malondialdehyde was affected by both treatment and sampling day (*P* < 0.01), as were protein carbonyls (*P* < 0.05). Specifically, colostrum of CON group showed higher content of malondialdehyde (*P* < 0.01) and protein carbonyls (*P* < 0.05) compared to ADD group.

**Table 10:** Oxidative profile of colostrum from dairy cows fed with 35g/day/head with (ADD; n = 10) or without (CON; n = 10) Oxyphenol® (*Pinus taeda* hydrolyzed lignin) from the beginning of the dry period through 60 days into lactation.

Parameter	Group^1^	Days *postpartum*					SEM^2^	Analysis of variance^3^		
		1	2	3	4	5		T	D	T × D
Malondialdehyde (μg/L)	CON	344.94^A^	267.36^B^	162.32^C^	125.32^CD^	87.13^D^	0.04	<0.001	<0.001	0.112
	ADD	228.72^a^	139.94^ab^	112.79^b^	100.26^b^	79.37^b^				
Protein carbonyls (nmol/mg protein)	CON	37.94	36.00^b^	39.41	41.22^a^	41.02	1.72	0.012	0.010	0.995
	ADD	34.70	33.12^b^	36.53	38.12^a^	39.21^a^				
FRAP^4^ (nmol ascorbic ac. eq./mL colostrum)	CON	209.88	211.39	227.18	203.01	208.8	17.99	0.154	0.626	0.918
	ADD	282.74	273.24	286.74	256.39	253.16				

A, B, C, D – Means with different superscripts differ significantly (*P* < 0.01) due to sampling day.

a, b – Means with different superscripts differ significantly (*P* < 0.05) due to sampling day.

1 CON = control treatment, ADD = Oxyphenol® treatment (*Pinus taeda* hydrolyzed lignin).

2 SEM = standard error of the mean.

3 T = treatment, D = sampling day, T × D = binary interaction between treatment and sampling day.

4 FRAP = Ferric reducing antioxidant power.

## 4 Discussion

The comprehensive evaluation of serum biochemical parameters in dairy cows revealed values that were predominantly within the physiological ranges described for the species (Piccione et al., 2012; Coroian et al., 2017). This overall stability suggests that Oxyphenol® did not adversely affect systemic health, as fluctuations in these biomarkers are widely recognized as sensitive indicators of organ functionality and metabolic homeostasis (Puppel & Kuczyńska, 2016). The onset of lactation is characterized by a sharp rise in the demand for key nutrients, such as glucose, amino acids, and fatty acids, to support milk synthesis. In high-yielding dairy cows, nutrient demand frequently exceeds dietary supply, leading to mobilization of body reserves and the development of negative energy balance that usually persist for several weeks after calving (Kessel et al., 2008). A key feature of this metabolic adaptation is the pronounced mobilization of NEFA from adipose tissue as early-lactation energy output rises faster than the concurrent ramp-up in feed intake. Despite a rapid postpartum increase in intake, this transient negative energy balance drives NEFA release; these preformed fatty acids are largely oxidized by the liver and contribute to milk-fat synthesis in the mammary gland. Kumprechtová et al. (2022) evaluated the effect of plant-derived bioactive compounds in the diet of transition dairy cows and observed a reduction in *postpartum* serum NEFA concentrations, suggesting decreased mobilization of adipose tissue and, consequently, a mitigated negative energy balance during the early lactation period. Previous studies have reported that NEFA concentrations often surpass the threshold of 0.6 mmol/L at calving, reflecting these adaptive processes (Vazquez-Anon et al., 1994; Cavestany et al., 2005; Seifi et al., 2007). In line with these findings, both groups in our study exhibited a transient rise in circulating NEFA at calving, followed by a rapid return to physiological values within one week postpartum, consistent with the observations of Vazquez-Anon et al., 1994 and Seifi et al., 2007. Notably, reductions in NEFA with specific flavonoids have also been reported more recently (e.g., naringin during the transition period improved systemic metabolic status and attenuated oxidative stress), reinforcing the plausibility of phenolic-driven mitigation of lipomobilization when bioavailability and dosing are adequate (Li et al., 2024). Epidemiologically, lower NEFA and BHB around calving are associated with reduced postpartum disease risk and better performance (Ospina et al., 2010; McArt et al., 2013). Parallel to the NEFA dynamics, plasma total cholesterol decreased at calving and increased during early lactation in both groups. This pattern agrees with the well-documented positive correlation between cholesterol concentrations and energy balance in early-lactation cows (Kida, 2002, Reist et al., 2003). The temporary decline observed around calving may indicate a limited capacity to cope with the heightened metabolic demands of this stage, whereas the subsequent rise in cholesterol could be explained by increased milk yield, dietary fat supplementation, or physiological adaptations occurring during early lactation (Vasilenko, 2020). Several studies have reported similar trajectories, with cholesterol levels declining in late gestation and rising markedly during the first months of lactation under standard feeding and management conditions (Seifi et al., 2007; Chalmeh et al., 2016; Folnožić et al., 2016, García et al., 2017). Mechanistically, the *postpartum* nadir and recovery of cholesterol likely reflect hepatic very-low-density lipoprotein export capacity and the resolution of negative energy balance. The experimental negative energy balance models demonstrate stage-dependent suppression of cholesterol metabolism with recovery as energy balance improves (Gross et al., 2015). In addition, inflammatory processes occurring around parturition may affect hepatic apolipoprotein synthesis and VLDL export, thereby contributing to changes in circulating cholesterol concentration. However, inflammatory markers were not measured in the present study, and this mechanism should therefore be considered only as a possible explanation. A significant effect of treatment was observed on hepatic-related enzymes, with ALT and ALP showing higher concentrations in CON compared with ADD. These findings may suggest a possible modulation of biochemical indicators associated with hepatic metabolism in Oxyphenol®-supplemented cows. However, this interpretation should be considered cautiously. ALT and ALP alone do not provide a complete assessment of liver function in transition dairy cows, and more specific indicators such as γ-glutamyl transferase, glutamate dehydrogenase, β-hydroxybutyrate, and inflammatory markers such as haptoglobin were not included in the present study. Therefore, the present results should be interpreted as changes in selected hepatic-related biochemical markers rather than as definitive evidence of improved liver health or reduced metabolic disease risk. Previous studies have reported that dietary polyphenols may modulate hepatic and metabolic responses in transition dairy cows. For example, Ma et al., 2021 observed that green tea polyphenols reduced serum concentrations of AST, ALT, and γ-glutamyl transferase in cows with hyperketonemia, whereas Stoldt et al., 2015 reported that intraduodenal quercetin affected postpartum liver-related indicators in periparturient cows. These findings support the biological plausibility that phenolic compounds may influence hepatic-related metabolism. Nevertheless, in the present study, the absence of more specific liver-function, ketone-body, and inflammatory markers prevents a definitive mechanistic interpretation. Future studies should include BHB, GGT, GLDH, haptoglobin, and postpartum clinical outcomes to clarify whether the observed biochemical changes translate into improved liver function or reduced incidence of transition disorders. However, the transition period is not only marked by metabolic adaptations but also by profound changes in oxidative balance, which can increase the susceptibility of cows to periparturient disorders (Bernabucci et al., 2005; Sordillo and Aitken, 2009). This makes antioxidant support particularly relevant, as conventional feeding systems largely focus on preventing deficiencies rather than optimizing redox status (Overton & Yasui, 2014). As discussed above, the redox trajectory around calving is closely related to energy balance and selected biochemical indicators. Therefore, oxidative markers should be interpreted together with NEFA, cholesterol, and liver-related enzymes, while acknowledging that the present metabolic profiling was not sufficient to fully characterize hepatic function or inflammatory status (Bernabucci et al., 2005; Sordillo and Aitken, 2009; Overton & Yasui, 2014). Against this background, the evaluation of oxidative markers in Oxyphenol®-supplemented cows highlights its potential role in mitigating oxidative stress. Specifically, serum levels of lipid peroxidation products were lower in the ADD treatment compared with CON during the *postpartum* period. This effect was most evident at +3 and +7 d *postpartum*, a window that typically shows the peak of circulating malondialdehydes/TBARS in dairy cows (Konvičná et al., 2015). Moreover, polyphenols-supplemented cows tended to exhibit reduced protein carbonyls concentrations and higher FRAP values relative to CON, although these differences did not reach statistical significance. Similar decreases in lipid peroxidation and improvements in plasma antioxidant capacity have been reported with plant polyphenol sources in transition cows (e.g., grape seed oil increased FRAP/TAC; pomegranate peel–based supplements modulated oxidative status) (Safari et al., 2018; Akhlaghi et al., 2022; Signor et al., 2024). These parallels support the biological plausibility that the phenolic mixture in Oxyphenol® can blunt postpartum oxidative pressure. With regard to the antioxidant defense system available to counteract oxidative insults, our analysis of enzymatic activity revealed lower SOD around calving and lower GSPx after calving in ADD than in CON. The lower SOD and GSPx activities observed in ADD cows, together with lower TBARS and reduced PBMC-derived ROS, may indicate a reduced need for compensatory enzymatic antioxidant activation because of a lower oxidative burden. However, this interpretation should be considered cautiously. Lower antioxidant enzyme activity may also reflect a reduced enzymatic defense response rather than exclusively an improved redox status. Therefore, SOD and GSPx activities should not be interpreted as isolated indicators, but rather in combination with lipid peroxidation markers, FRAP values, and PBMC ROS production. In this context, the overall pattern observed in ADD cows suggests an attenuation of oxidative pressure around calving, although the mechanisms underlying the modulation of antioxidant enzyme activity require further investigation. Consistently, FRAP showed a numerical rise in ADD at +30 and +60 d, which may further support the interpretation of an improved redox balance, although this finding should be considered together with the other oxidative and cellular markers (↓TNF-α, ↓ROS/RNS, ↑cell viability) (Abuelo et al., 2015; Konvičná et al., 2015; Ciliberti et al., 2020). Finally, the redox profile observed in ADD cows coincided with lower ALT and ALP concentrations, supporting a coherent pattern of improved oxidative and biochemical indicators around calving, while acknowledging that the present metabolic profiling was not sufficient to fully characterize liver function or inflammatory status (Bernabucci et al., 2005; Abuelo et al., 2015; Abuelo et al., 2019; Ma et al., 2021). Consistently, the assessment of ROS production by PBMC supported this interpretation. Cells from the CON treatment showed higher intracellular ROS levels compared with those from ADD cows, indicating a greater oxidative burden and, consequently, a higher demand for antioxidant enzyme activity. Conversely, the reduced ROS synthesis observed in PBMC from Oxyphenol®-supplemented animals suggests that the treatment may have mitigated oxidative processes at the cellular level, contributing to a more balanced redox state and improved cellular response to oxidative stress (Maggiolino et al., 2024). Excessive generation of ROS, when not adequately counterbalanced by antioxidant defenses, can lead to cytotoxic events and oxidative damage to key cellular components such as lipids, proteins, and nucleic acids (Ciampi et al., 2020; Dinardo et al., 2022). Such oxidative imbalance is particularly critical during the peripartum period, when the increased metabolic demand and immune activation make cells more vulnerable to oxidative stress (Mutinati et al., 2014). In our study, the between-group gap in PBMC-derived ROS was most evident from 7 days prepartum to 3 days postpartum, paralleling the window in which plasma TBARS also diverged, thereby reinforcing the systemic–cellular concordance of the ADD effect. This is biologically plausible because periparturient immunosuppression and redox dysregulation are tightly intertwined in dairy cows (Sordillo and Aitken, 2009; Abuelo et al., 2015). In this context, the evaluation of PBMC viability provided additional insights into the potential protective role of Oxyphenol®. During the tight periparturient phase (from 3 days before to 3 days after calving), PBMCs from cows supplemented with Oxyphenol® showed higher cell viability compared with the control group, even following mitogenic stimulation with conA. Although H_2_O_2_ exposure markedly reduced cell viability in both groups, as expected given its strong pro-oxidant effect jeopardizing SOD activity, PBMC from the ADD treatment maintained significantly higher survival rates at calving. Notably, the ADD–CON differences were most pronounced at calving and persisted to +3 d under ConA stimulation, whereas under H₂O₂ challenge the advantage for ADD was evident at calving (Fig. 1, Fig. 2). These results are consistent with prior work showing that phenolic supplementation can attenuate immune-cell oxidative responses and sustain ex vivo functionality in cattle; for example, hydrolyzed lignin from *Pinus taeda* reduced ROS and pro-inflammatory readouts while improving PBMC viability (Ciliberti et al., 2020), and naringin improved systemic oxidative status during transition (Li et al., 2024). At the mechanistic level, phenolics abundant in Oxyphenol® (quercetin/rutin-type flavonols, eriodictyol, rosmarinic- and methyl-gallate derivatives) are known to activate Nrf2 and temper NF-κB, limiting mitochondrial and NADPH-oxidase–derived ROS and thereby preserving lymphocyte proliferation under mitogenic or oxidative stimuli (Song et al., 2014, Song et al., 2016). These cellular findings are consistent with the plasma oxidative profile and support the interpretation that Oxyphenol® supplementation was associated with a more favorable redox status around calving. Finally, because NEFA and BHB can directly induce ROS-mediated injury in bovine hepatocytes and immune cells, the simultaneous mitigation of lipomobilization markers and PBMC oxidative activity observed here is coherent with an immunometabolic mode of action for polyphenols in transition cows (Sordillo and Aitken, 2009; Song et al., 2014; Abuelo et al., 2015).

Alongside their role in health and oxidative status of the animals, the inclusion of polyphenols in the diet of dairy cows has gained increasing attention for their potential impact on productive performance and milk quality (O’connell and Fox, 2001). Beyond their well-documented antioxidant and anti-inflammatory properties, polyphenols may be transferred into milk, potentially enhancing its nutritional and functional value. However, in contrast to monogastric species, the transfer of polyphenolic compounds from feed to milk in ruminants is considerably more complex (Gessner et al., 2017). Within the rumen, polyphenols undergo extensive biotransformation through microbial metabolism, followed by additional modifications in the liver and other tissues (Wein et al., 2016). Consequently, the compounds ultimately secreted into milk may differ substantially from those originally ingested, both in chemical structure and biological activity (Rocchetti et al., 2022). Consistent with this, targeted metabolomics in ruminants shows the appearance in milk of phase-II conjugates and microbial derivatives (e.g., phenylpropionic/hippuric-type metabolites), rather than the parent flavonoids, with concentrations depending on dose, matrix, and stage of lactation (Gessner et al., 2017; Rocchetti et al., 2022). Understanding these processes is therefore essential to elucidate how dietary polyphenols influence ruminal fermentation, systemic metabolism, and mammary gland function, ultimately shaping milk yield and composition. Nevertheless, a growing body of literature supports the transfer of dietary polyphenols or their metabolites into ruminant milk, or at least improvements in milk quality parameters attributed to polyphenol supplementation, either through their direct inclusion or via polyphenol-rich by-products (Formato et al., 2022, Serra et al., 2021). For instance, Cohen-Zinder et al., 2017 observed higher milk yield and antioxidant capacity, along with lower somatic cell counts, in Holstein cows fed *Moringa oleifera* silage (270 g/kg DM) from three weeks before calving until six weeks *postpartum*. Similarly, Delgadillo-Puga et al., 2019 found that the inclusion of *Acacia farnesiana* pods in the diet of goats increased the polyphenol content and antioxidant activity of milk, while reducing its cholesterol concentration. Moreover, Moate et al., 2014 observed an increase in concentrations of MUFAs, PUFAs, and cis-9, trans-11 linoleic acid in milk from cows supplemented with either dried or ensiled grape marc. Other polyphenol-rich feeds (e.g., olive by-products, grape pomace) have likewise improved milk antioxidant capacity and/or oxidative stability, sometimes without altering gross composition, suggesting that redox effects may occur independently of major shifts in fat, protein, or lactose (Castellani et al., 2017; Vargas-Bello-Pérez et al., 2018). In the present study, Oxyphenol® supplementation did not significantly affect milk chemical composition, coagulation properties, or fatty acid profile. This lack of change in rennet coagulation time and curd firmness is plausible given that ruminal biotransformation likely limited direct casein–polyphenol interactions at the udder level (O’connell and Fox, 2001; Malacarne et al., 2014). However, a distinct effect emerged in the oxidative profile, as milk from ADD cows exhibited higher antioxidant potential and lower concentrations of lipid oxidation products. Importantly, these milk outcomes mirrored the systemic findings (lower plasma TBARS and PBMC-derived ROS in ADD), supporting a system-to-milk linkage whereby improved maternal redox status translates into enhanced oxidative stability of milk (Sordillo and Aitken, 2009; Abuelo et al., 2015). Given that the milk fatty-acid profile did not differ between groups, the reduced formation of secondary lipid-oxidation products in ADD is unlikely to be explained by a shift toward less oxidizable lipids. Rather, these findings are compatible with improved mammary redox status and, as a hypothesis requiring confirmation, possible carryover of antioxidant metabolites into milk (Gessner et al., 2017; Rocchetti et al., 2022). This finding is particularly noteworthy because it indicates improved oxidative stability of milk from Oxyphenol®-supplemented cows. However, because phenolic compounds or their metabolites were not directly quantified in plasma or milk, these results cannot be considered direct evidence of transfer from feed to milk. Rather, they are compatible with the hypothesis that Oxyphenol® supplementation improved milk oxidative stability through systemic redox modulation and/or possible carryover of antioxidant metabolites into mammary secretions. It should also be noted that the transfer and bioavailability of dietary polyphenols in ruminants depend on several interacting factors, including the chemical structure of the compounds, their inclusion level, the dietary matrix, rumen microbial metabolism, and the physiological status of the host (Serra et al., 2021; Forte et al., 2026). Therefore, future studies should combine targeted or untargeted UHPLC-HRMS metabolomic profiling of plasma and milk with pharmacokinetic sampling across lactation to identify Oxyphenol®-derived metabolites, quantify their potential carryover, and relate them to oxidative markers and technological traits. Although the literature on colostrum is comparatively limited, a growing body of evidence supports the transfer of dietary polyphenols or their metabolites into ruminant secretions, or at least improvements in product oxidative stability with polyphenol-rich feeds (Formato et al., 2022, Serra et al., 2021). For instance, supplementation with chestnut tannins in dairy cows during the peripartum period has been associated with improvements in colostrum quality (e.g., higher IgG) and antioxidant status (Prodanović et al., 2021). These heterogeneous outcomes align with the upstream constraints imposed by rumen metabolism, matrix effects, and stage of lactation (Rolinec et al., 2021). In our study, the chemical composition of colostrum was not affected by Oxyphenol® supplementation during the *peripartum* period. A decrease in DM content was observed, likely due to the natural decline in protein concentrations during the first days after calving. This reduction in colostral protein is a well-documented physiological process, as the concentration of immunoglobulins (Ig), particularly IgG, decreases markedly with each passing hour after parturition. For this reason, among others, to ensure optimal passive immune transfer, colostrum should be ingested within the first two hours after birth (Ahmann et al., 2021; Röder et al., 2023). To contextualize these targets with standardized cut-points, high-quality first-milking colostrum is commonly defined as IgG ≥ 50 g/L, which corresponds to a Brix threshold of ≥ 22% (McGuirk and Collins, 2004; Godden, 2008; Bielmann et al., 2010; Sockett et al., 2023). In our data, day-1 Brix in ADD averaged slightly above this threshold versus CON (22.85 vs. 21.71), although not statistically different, which is consistent with biological variability and the steep early postpartum decline in IgG. (McGuirk and Collins, 2004), (Bielmann et al., 2010). Interestingly, while Oxyphenol® supplementation did not alter the overall chemical composition of colostrum, it appeared to influence its oxidative profile. In particular, cows receiving Oxyphenol® showed reduced levels of lipid oxidation products in colostrum, suggesting improved oxidative stability of early mammary secretions during the postpartum period. These colostrum findings mirror the systemic patterns observed in supplemented cows, including lower plasma TBARS and reduced PBMC-derived ROS, and are therefore consistent with an improvement in maternal and mammary redox status. However, because phenolic compounds or their metabolites were not directly quantified in maternal plasma, colostrum, or calf plasma, the present results do not demonstrate transfer of antioxidant molecules to the newborn. This possibility should be considered a hypothesis rather than a demonstrated mechanism. Future studies should include metabolomic profiling of maternal plasma, colostrum, and calf plasma, together with indicators of passive immune transfer and neonatal oxidative status, to clarify whether Oxyphenol®-derived metabolites can reach colostrum and whether this has biological relevance for calves.

## 5 Study limitations

The present study has some limitations that should be acknowledged. First, although the number of animals was determined by an a priori power analysis, the sample size was limited, and transition-cow responses are characterized by high biological variability. Factors such as body condition score, previous milk yield, calving difficulty, and individual metabolic status may influence oxidative and metabolic responses and should be considered in future larger-scale studies. Second, the metabolic profile did not include some key transition-cow indicators, such as β-hydroxybutyrate, γ-glutamyl transferase, glutamate dehydrogenase, and haptoglobin, which would have strengthened the interpretation of liver function, inflammatory status, and metabolic stress. Therefore, the lower ALT and ALP concentrations observed in supplemented cows should be interpreted as changes in selected hepatic-related biochemical indicators rather than as definitive evidence of improved liver health. Third, postpartum clinical outcomes, including ketosis, retained placenta, metritis, mastitis, and displaced abomasum, were not systematically recorded. Consequently, the practical relevance of the present findings should be interpreted mainly in relation to biochemical, oxidative, and cellular indicators rather than direct evidence of reduced clinical disease incidence. Finally, phenolic compounds or their metabolites were not directly quantified in plasma, milk, or colostrum. Therefore, the observed improvement in milk and colostrum oxidative stability should be interpreted as compatible with, but not demonstrative of, phenolic metabolite transfer into mammary secretions. Future studies should combine larger cow populations, postpartum clinical monitoring, and targeted metabolomic profiling to clarify the mechanisms underlying the effects of Oxyphenol® supplementation during the transition period.

## 6 Conclusions

The present study demonstrated that dietary supplementation with Oxyphenol® during the *peripartum* period did not alter the systemic metabolic profile, milk yield, or chemical composition of colostrum and milk, indicating that the treatment was well tolerated and did not interfere with the physiological adaptations occurring around calving. Concurrently, cows receiving Oxyphenol® showed a consistently improved redox phenotype, lower plasma TBARS with blunted SOD/GSPx up-regulation, reduced PBMC-derived ROS with higher peripartum viability, and lower milk/colostrum lipid-oxidation products (with numerically higher FRAP), together with lower ALT/ALP, suggesting a more favorable systemic and mammary redox profile during the calving transition. These findings suggest improved oxidative stability of mammary secretions in supplemented cows. However, because phenolic compounds or their metabolites were not directly quantified, the possible transfer of Oxyphenol®-derived metabolites into milk or colostrum remains a hypothesis that requires confirmation through targeted metabolomic studies. From a product perspective, this may translate into technological and shelf-life advantages without compromising processing traits. Further studies including larger cow populations, postpartum clinical outcomes, and targeted metabolomic profiling of plasma, milk, and colostrum are needed to confirm these mechanisms and clarify the practical relevance of Oxyphenol® supplementation in transition dairy cows.

## Statement for studies in humans/animals

This trial was approved by the Ethics Committee of the Department of Veterinary Medicine, University of Bari (protocol no. 07/2023).

## Funding

This study received no external funding.

## CRediT authorship contribution statement

**Lucrezia Forte:** Writing – original draft, Visualization, Software, Formal analysis, Data curation. **Pasquale De Palo:** Writing – original draft, Visualization, Validation, Conceptualization. **Elisabetta Casalino:** Writing – review & editing, Methodology, Investigation. **Tiziana Latronico:** Writing – review & editing, Methodology, Investigation. **Grazia Maria Liuzzi:** Writing – review & editing, Validation. **Aristide Maggiolino:** Writing – original draft, Supervision, Resources, Project administration, Funding acquisition.

## Declaration of competing interest

The authors declare that they have no known competing financial interests or personal relationships that could have appeared to influence the work reported in this paper.
